# Correlative Imaging of Individual CsPbBr_3_ Nanocrystals: Role of Isolated Grains in Photoluminescence of Perovskite Polycrystalline Thin Films

**DOI:** 10.1021/acs.jpcc.3c03056

**Published:** 2023-06-20

**Authors:** Petr Liška, Tomáš Musálek, Tomáš Šamořil, Matouš Kratochvíl, Radovan Matula, Michal Horák, Matěj Nedvěd, Jakub Urban, Jakub Planer, Katarína Rovenská, Petr Dvořák, Miroslav Kolíbal, Vlastimil Křápek, Radek Kalousek, Tomáš Šikola

**Affiliations:** † Institute of Physical Engineering, Faculty of Mechanical Engineering, Brno University of Technology, Technická 2896/2, 616 69 Brno, Czech Republic; ‡ Central European Institute of Technology, Brno University of Technology, Purkyňova 123, 612 00 Brno, Czech Republic; § Faculty of Chemistry, Brno University of Technology, Purkyňova 464/118, 612 00 Brno, Czech Republic; ⊥ Tescan Orsay Holding, a.s, Libušina tř. 21, Brno 623 00, Czech Republic

## Abstract

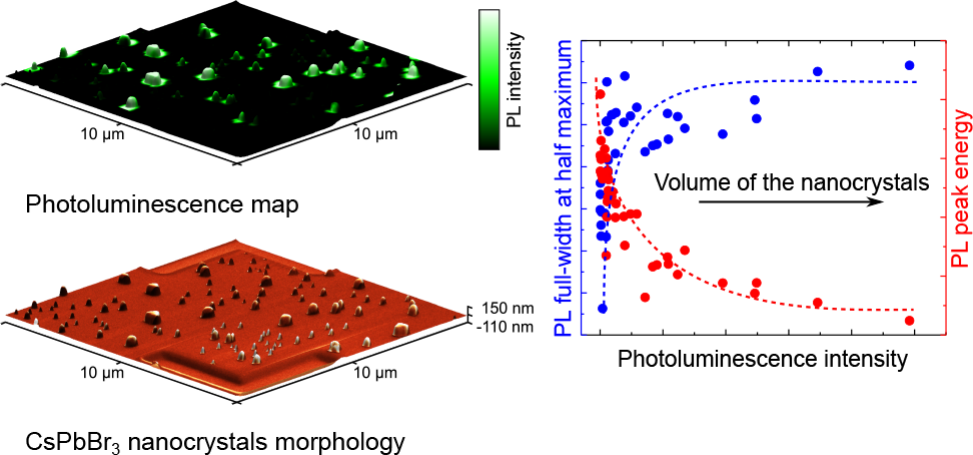

We report on the optical properties of a CsPbBr_3_ polycrystalline thin film on a single grain level. A sample composed of isolated nanocrystals (NCs) mimicking the properties of the polycrystalline thin film grains that can be individually probed by photoluminescence spectroscopy was prepared. These NCs were analyzed using correlative microscopy allowing the examination of structural, chemical, and optical properties from identical sites. Our results show that the stoichiometry of the CsPbBr_3_ NCs is uniform and independent of the NCs’ morphology. The photoluminescence (PL) peak emission wavelength is slightly dependent on the dimensions of NCs, with a blue shift up to 9 nm for the smallest analyzed NCs. The magnitude of the blueshift is smaller than the emission line width, thus detectable only by high-resolution PL mapping. By comparing the emission energies obtained from the experiment and a rigorous effective mass model, we can fully attribute the observed variations to the size-dependent quantum confinement effect.

## Introduction

Fully inorganic lead halide perovskite (LHP) CsPbBr_3_ is a semiconducting material with a direct band gap exhibiting unique optical properties such as high internal and external quantum yields of both Stokes and anti-Stokes photoluminescence (PL), narrow PL line width, low nonradiative losses, remarkable photostability, and high PL emission wavelength tunability due to a strong quantum confinement effect (QCE).^1−9^ For its unique optical properties, CsPbBr_3_ has emerged as a contemporary material that has fuelled further intensive development in the fields of optoelectronics such as light-emitting devices,^10,11^ single photon-emitters,^12^ photovoltaics,^13−15^ high-energy γ radiation detectors,^16^ photocatalysis of chemical processes,^17^ high-resolution displays,^18^ nonlinear optical wavelength converters,^19^ reconfigurable memristors,^20^ hyper-sensitive scintillators,^21−23^ or even functional metasurfaces.^24^ The exceptionality of CsPbBr_3_ originates from the unique electronic fine structure and high tolerance toward structural defects.^25,26^ Moreover, due to the inherent ionic character of CsPbBr_3_, a cheap and facile fabrication of CsPbBr_3_ nanocrystals (NCs) is possible simply by mixing the corresponding precursor solutions without the need for elevated temperature or other, potentially challenging conditions.^27^ Hence, NCs and their thin films in general form the basis of halide perovskite-based optoelectronic devices with unique or improved optical and electronic properties.^28^ The optical and electronic properties of CsPbBr_3_ NCs are strongly dependent on their dimensions, morphology, size distribution, and surface passivation.^29,30^ Decreasing the size of CsPbBr_3_ crystals to the nanoscale or appropriate surface passivation can be utilized to tune or even enhance their optical properties.^31−36^ Full control over the size of the colloidal lead halide perovskite nanocrystals (cn-LHP) and its distribution has been demonstrated by Protesescu et al., who presented a hot-injection method allowing the colloidal synthesis of well-defined, monodisperse, and monocrystalline NCs with PL properties far exceeding the polycrystalline films (pf-LHP).^37^

However, cn-CsPbBr_3_ prepared by the hot-injection method suffer from a significant underperformance due to problems with electrical contacting which substantially limits their usage in optoelectronic devices in comparison to their polycrystalline film (pf-CsPbBr_3_) counterparts.^38^ Therefore, the prospect of long operational devices stability, high conversion efficiency (25%), and simple utilization in electroluminescent devices make pf-CsPbBr_3_ better candidates for today’s optoelectronic devices than cn-CsPbBr_3_, especially for solar cells technology.^38−41^

A suitable way to study the optical properties of pf-CsPbBr_3_ such as the emission wavelength, spectral broadening, recombination kinetics processes, and internal electrochemical potential of free charge carriers is through PL spectroscopy.^1,42−46^ When correlated with the high-resolution imaging techniques of low-dimensional structures, PL spectroscopy provides a direct insight into the influence of local morphology effects^47^ or the energy levels shift caused by QCE.^48−50^ However, due to the limited spatial resolution of optical microscopy, this approach cannot provide a response of individual grains of a polycrystalline thin film, as there are usually several grains within the focus.

Here, we demonstrate a comprehensive analysis of the material properties of individual CsPbBr_3_ NCs prepared from a low-concentration pf-LHPs solution while achieving considerable spacing between individual NCs. This system is representative for thin polycrystalline films, as it was grown under the conditions typically used for the growth of thin films. In contrast to the thin polycrystalline film with overlapping grains, the well-isolated NCs can be addressed individually by optical spectroscopy. A correlative approach based on focused ion beam (FIB) tagging of the examined area on the sample is used and the synergy of high-resolution experimental techniques is utilized: transmission electron microscopy (TEM), scanning electron microscopy (SEM), atomic force microscopy (AFM), and confocal optical spectroscopy (COS) together with anti-Stokes PL mapping. These techniques are capable of analyzing CsPbBr_3_ NCs’ inner structure, determining their characteristic dimensions, morphology, and their PL response. We experimentally retrieve the dependence of the PL peak emission wavelength on the characteristic volume and aspect ratio of the NC at a single NC level. We demonstrate that the emission energy is governed by a size-dependent QCE predictable within a simple effective mass model. An understanding of the relation between the characteristic dimension of individual CsPbBr_3_ NCs and their emission energy is a crucial element in their implementation in advanced optoelectronic devices.

## Methods

### Fabrication of CsPbBr_3_ NCs

Transparent fused silica substrates covered by an indium–tin-oxide (ITO) layer with a thickness of 50 nm were subsequently cleaned by acetone (5 min), isopropyl alcohol (5 min), and deionized water (5 min) baths in ultrasound. After the deionized water treatment, the residues of water and dust particles were blown off with nitrogen and by placing the substrates on a hot-plate (100 °C) for 10 min. For the synthesis of CsPbBr_3_ NCs, a saturated solution of CsBr and PbBr_2_ precursors dissolved in dimethylformamide (DMF) was dropcasted onto the substrates and left to dry out at room temperature.

### Correlative Imaging of CsPbBr_3_ NCs

The FIB micromarkings for navigation across the sample and correlative imaging were fabricated using a focused ion beam/scanning electron microscope TESCAN LYRA3. The energy of Ga^+^ primary ions was 30 keV, ion beam current 2 nA, and the target depth of the micromarkings was an equivalent of 200 nm in a Si substrate.

The morphology of the CsPbBr_3_ NCs was obtained by subsequent analysis by SEM and AFM. The SEM measurements were performed using high-resolution SEM FEI Verios 460L at 3 kV and 13 pA. The AFM measurements were performed using a scanning probe microscope Bruker Dimension Icon with ScanAsyst-Air high-resolution imaging probes with a triangular geometry and a tip radius of 12 nm.

The optical properties of the individual CsPbBr_3_ NCs were measured by PL spectroscopy. The PL maps were obtained with a Witec Alpha 300R confocal microscopy and optical spectroscopy system. The laser light with excitation wavelength of 532 nm and line width of 1 nm was used. The observed PL spectra are of single-photon phonon-assisted anti-Stokes PL origin and nearly identical to the PL spectra with Stokes origin. As has been demonstrated, both the Stokes and anti-Stokes excitation yield nearly identical PL spectra.^51^ The obtained PL maps have a resolution of 120 × 120 pixels^2^, and they were obtained with an objective Zeiss EC Epiplan-Neofluar DIC with 100× magnification and numerical aperture of 0.9 and 600 grating/mm diffraction grid. The spectra were collected by utilizing the laser light with optical power of 37 μW and integration time of 0.1 s. The parameters *I*_0_ (PL peak intensity), λ_0_ (PL peak emission wavelength), and λ_fwhm_ (PL full width-at-half-maximum (fwhm)) were obtained by fitting the Gaussian function to the background-subtracted PL spectra:
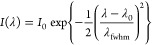
1

Chemical imaging was performed by time-of-flight secondary ion mass spectrometry (ToF-SIMS) and X-ray photoelectron spectroscopy (XPS) instrumentations. The ToF-SIMS measurements were performed using a TESCAN AMBER focused ion beam-scanning electron microscope (FIB-SEM) equipped with an orthogonal ToF-SIMS system (C-TOF module provided by TOFWERK). The energy of Ga^+^ primary ions was 30 keV, ion beam current 47 pA, and pixel dwell time 11 μs. The Br 3D chemical image was done in the negative ion mode, and Ga, Cs, and Pb maps were obtained in the positive ion mode. The XPS measurements were performed using an X-ray photoelectron spectroscopy axis supra (KRATOS-XPS). The XPS spectra were fitted using a Lorenz curve and U2 Tougaard background subtraction. The orbitals used for determining the stoichiometry were Cs 3d_3/2_, Cs 3d_5/2_, Pb 4f_5/2_, Pb 4f_7/2_, Br 3d_3/2_, and Br 3d_5/2_.

Structural analysis of CsPbBr_3_ NCs was carried out by high-resolution TEM analysis. TEM, STEM, and electron energy loss spectroscopy (EELS) measurements were conducted by a (scanning) transmission electron microscope FEI Titan Themis. High-resolution imaging was performed in the TEM mode at 300 keV. STEM imaging and EELS were performed in a monochromated scanning regime at 120 keV, while the convergence semiangle was set to 10 mrad and the collection semiangle for EELS set to 14.4 mrad.

### Density Functional and Effective Mass Theory

As a starting point for the CsPbBr_3_ elementary cell volume relaxation, which enabled the theoretical computation of the CsPbBr_3_ electronic structure and further structural analysis, the experimentally obtained lattice parameter *a* = 5.9 Å  was used. The calculation at fixed volume utilized the lattice parameter, which varied within *a* ± 7%. By conducting volume relaxation of the elementary cell, it is possible to retrieve the dependence of the free energy per unit cell *F* on the volume of the CsPbBr_3_ elementary cell *V*. The minimum of the *F*(*V*) function corresponds to the equilibrium elementary cell volume *V*_0_ and thus to the equilibrium lattice constant *a*_0_, obtained by fitting *F*(*V*) by the Murnaghan equation of state
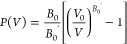
2where *P* is the pressure acting on the unit cell acquired by the partial derivation *P*(*V*) = −(∂*F*/∂*V*)_T, N_ at a constant temperature *T* and with a constant number of particles in the enclosed system *N*. The calculated lattice parameter was utilized in density functional theory (DFT) calculations of the electronic structure of CsPbBr_3_.

All DFT calculations were performed with the Vienna ab initio Simulation Package (VASP)^52−57^ using the projector-augmented wave method^56^ for treating core electrons. The Bloch functions for nine valence electrons of cesium (5s^2^5p^6^6s^1^), 14 valence electrons of lead (6s^2^5d^10^6p^2^), and seven valence electrons of bromine (2s^2^5p^5^) were expanded in a plane wave basis set with an energy cutoff 400 eV. The Brillouin zone was sampled with a Gamma-centered 6 × 6 × 6 Monkhorst–Pack grid.^58^ The self-consistent electronic calculations converged to 10^–5^ eV. Spin–orbit coupling was taken into account in all calculations. We used the PBEsol functional^59^ for geometry optimization of the cubic CsPbBr_3_. The electronic band structure of the cubic CsPbBr_3_ was calculated with a modified HSE06 functional^60^ with 45% Hartree–Fock mixing. The graph of the electron structure and the values of effective masses were retrieved with the help of the program Vaspkit.^61^ The effective masses were obtained by a third-order polynomial fit of the energy bands.

The electronic band gap of CsPbBr_3_*E*_g_ = 2.425 eV used in the effective mass theory calculations was determined by fitting the experimental data with the theoretical model (eq 4). The binding energy of an exciton in CsPbBr_3_ was determined by the hydrogen atom model
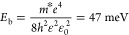
3where m* = 0.08*m*_e_ and ε = 4.8 is the experimental permittivity value for CsPbBr_3_.^25^

## Results and Discussion

**Figure 1:**
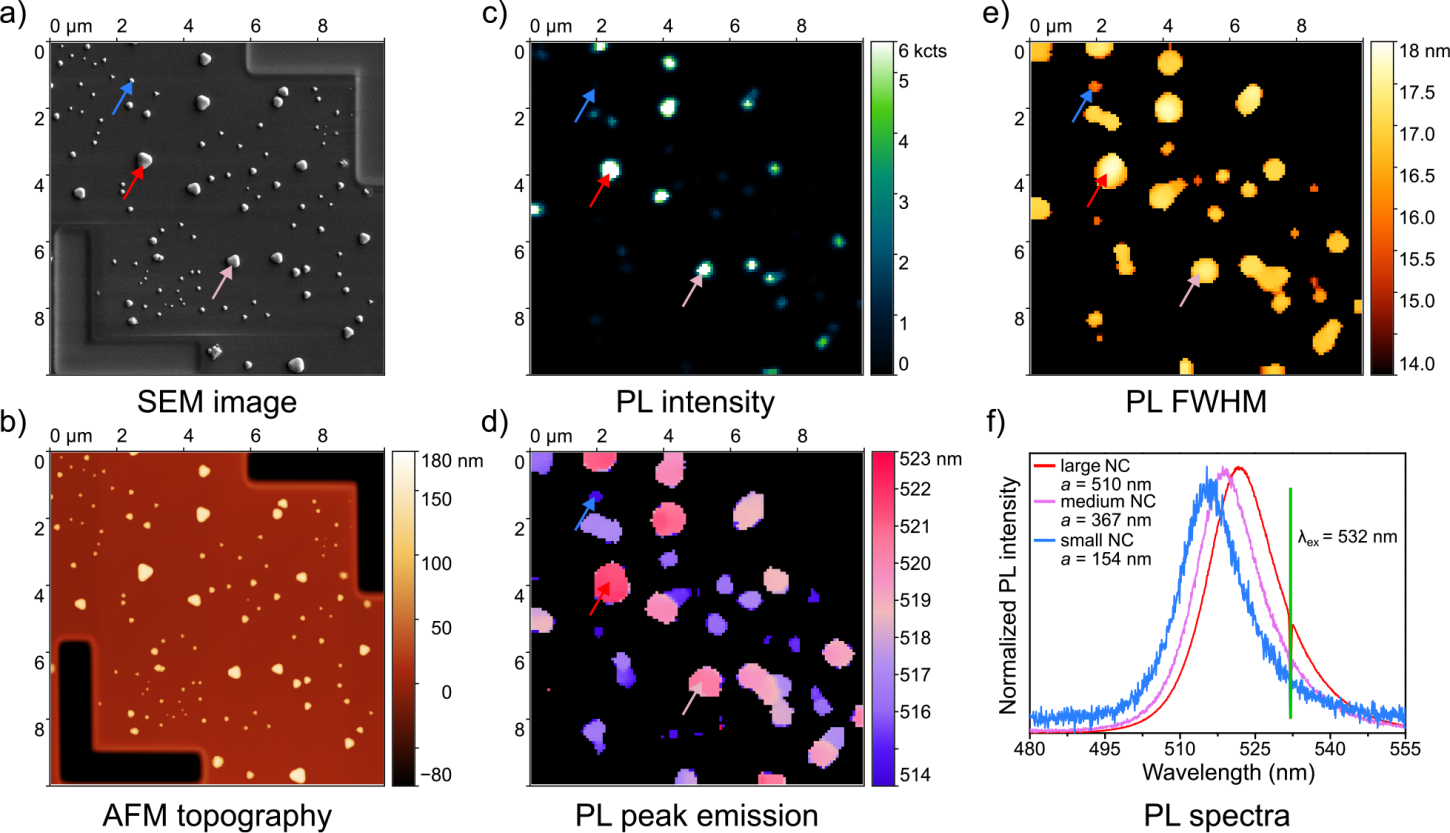
a) SEM image showing the CsPbBr_3_ NCs of various sizes and shapes. b) AFM topography image of the identical area displaying the height profile of the NCs. c) PL intensity, d) PL peak emission wavelength, and e) PL fwhm maps acquired by measuring PL spectra in the examined area and regression analysis of the background-corrected PL spectra. f) Representative PL spectra of three CsPbBr_3_ NCs marked by the arrows in the SEM image and PL maps. The characteristic widths *a* are indicated for each of the NCs.

Figure 1a shows an SEM image of the sample with CsPbBr_3_ NCs and FIB micromarkings used for the navigation across the sample and correlation of the obtained data. Here, we observe NCs of various shapes and sizes with the size distribution ranging from tens to hundreds of nm with the most frequent values peaking around 120 nm. The SEM image is accompanied by the AFM topography image (Figure 1b) of the identical sample area, which displays the height distribution of the NCs ranging from 10^1^ to 10^2^ nm. Both SEM and AFM images are utilized to determine the aspect ratio *AR* = *a*/*c* and the volume *V* of NCs, based on the characteristic width *a* and height *c* that are measured by SEM and AFM, respectively.

The morphology is correlated to PL maps in Figure 1c–e. The PL maps were obtained by COS (every pixel of the PL map has an assigned PL spectrum) and subsequent regression analysis of the PL spectra (see Methods). In Figure 1c, we see the PL intensity map that matches the positions of the CsPbBr_3_ NCs visible in Figure 1a and b. By comparing these images, it is evident that bigger NCs exhibit brighter PL. Figure 1d displays the PL peak emission wavelength of the NCs ranging from 514 nm (2.41 eV) for the smaller NCs to 523 nm (2.37 eV) for the bigger NCs. The PL fwhm map (Figure 1e) shows values ranging from 14 nm (68 meV) for smaller NCs to 18 nm (80 meV) for the bigger ones. Thanks to the correlation of structural (Figure 1a,b) and optical maps (Figure 1c,d,e), we were able to assign particular PL spectra to individual CsPbBr_3_ NCs (Figure 1f).

**Figure 2:**
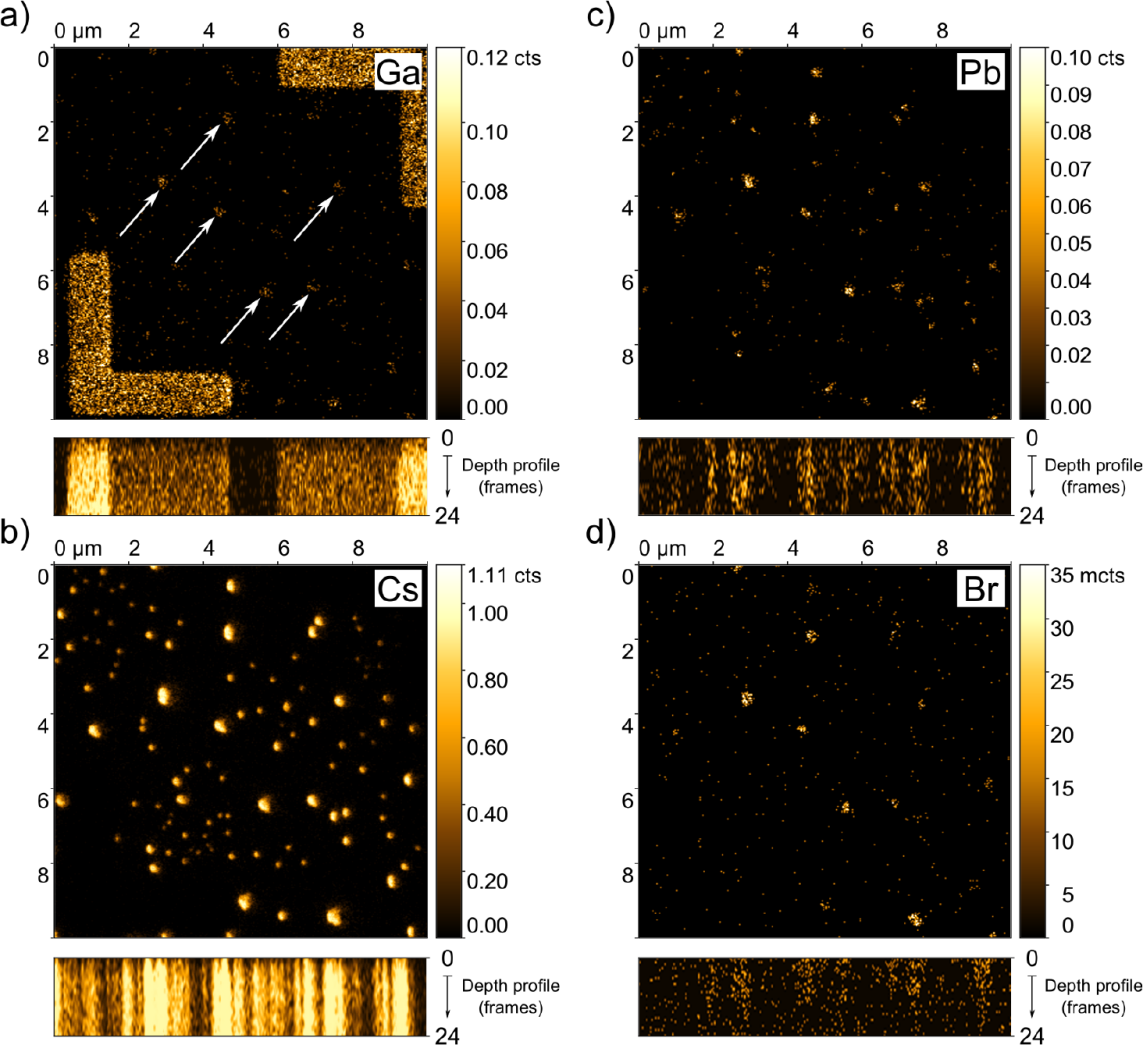
Analyzed region on the sample used for the 3D elemental mapping of a) Ga, b) Cs, c) Pb, and d) Br atoms. The white arrows in part a show the Ga ion implementation into the CsPbBr_3_ NCs. The intensity of the signal obtained by ToF-SIMS in b–d does not indicate any inhomogeneity in the spatial or depth elemental distribution. This assumption is based on a comparison of ToF-SIMS signals between NCs with similar volumes.

**Figure 3:**
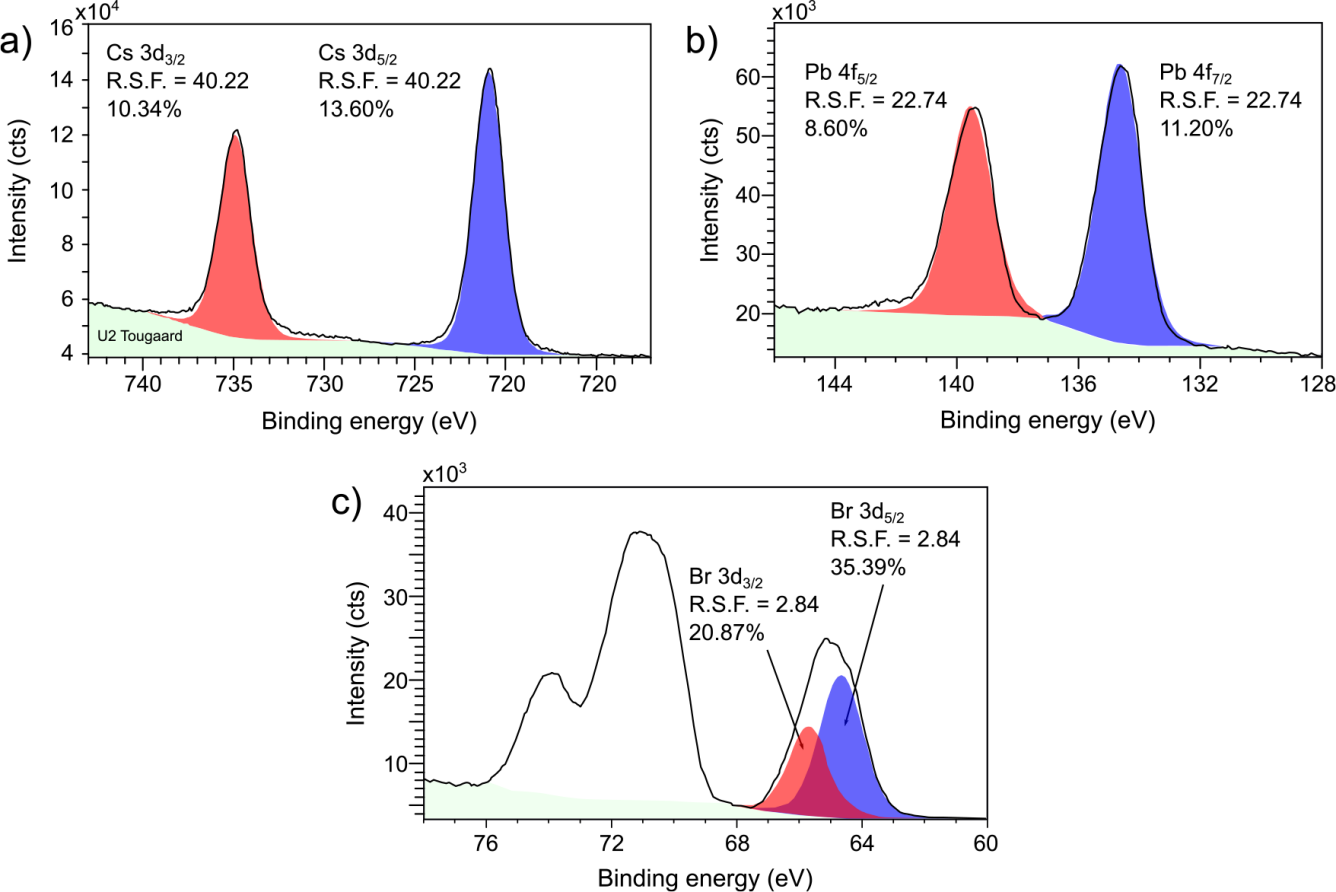
XPS spectra for determination of the stoichiometry of a) Cs, b) Pb, and c) Br atoms.

To exclude the role of local stoichiometry on the variance of PL properties of individual NCs, correlative elemental imaging by ToF-SIMS was performed. In Figure 2a–d, an analyzed area is shown (the same as in Figure 1) to which is correlated the 3D spatial distributions of the detectable elements: Ga, Cs, Pb, and Br ions acquired via ToF-SIMS analysis. Since Ga ion beam was used for both FIB micromarking and ToF-SIMS elemental imaging, it is expected that the Ga ions will be present on the sample surface. From Figure 2a, it seems that some Ga ions were implanted also inside CsPbBr_3_ NCs (see the white arrows in Figure 2a). The fabrication of FIB micromarkings could in principle lead to the alteration of structural and optical properties of the examined samples. The influence of Ga ions implanted during the fabrication of FIB micromarkings on the optical properties of CsPbBr_3_ NCs was studied as follows. The PL response of several NCs was measured before and after their exposure to gallium ions used for fabrication of the FIB markings. We discovered that the exposure to Ga ions of applied dose decreases the PL integral intensity by about 6% but does not alter the PL peak emission wavelength or fwhm (see Supporting Information S1, Figure S1). Figure 2b–d demonstrate the identical distribution of Cs, Pb, and Br elements throughout the whole volume of the sample. However, we were not able to determine the stoichiometry of individual NCs since ToF-SIMS is a qualitative rather than quantitative analysis. Therefore, the stoichiometry of the entire NCs ensemble was determined by integral XPS (Figure 3) as Cs_1.2_Pb_1_Br_2.8_. This stoichiometry, even though not being exact, points to high chemical quality of the measured NCs. These findings indicate, that variations observed in the PL peak emission wavelength might be attributed to the morphology differences of the individual NCs and the related QCE.

**Figure 4:**
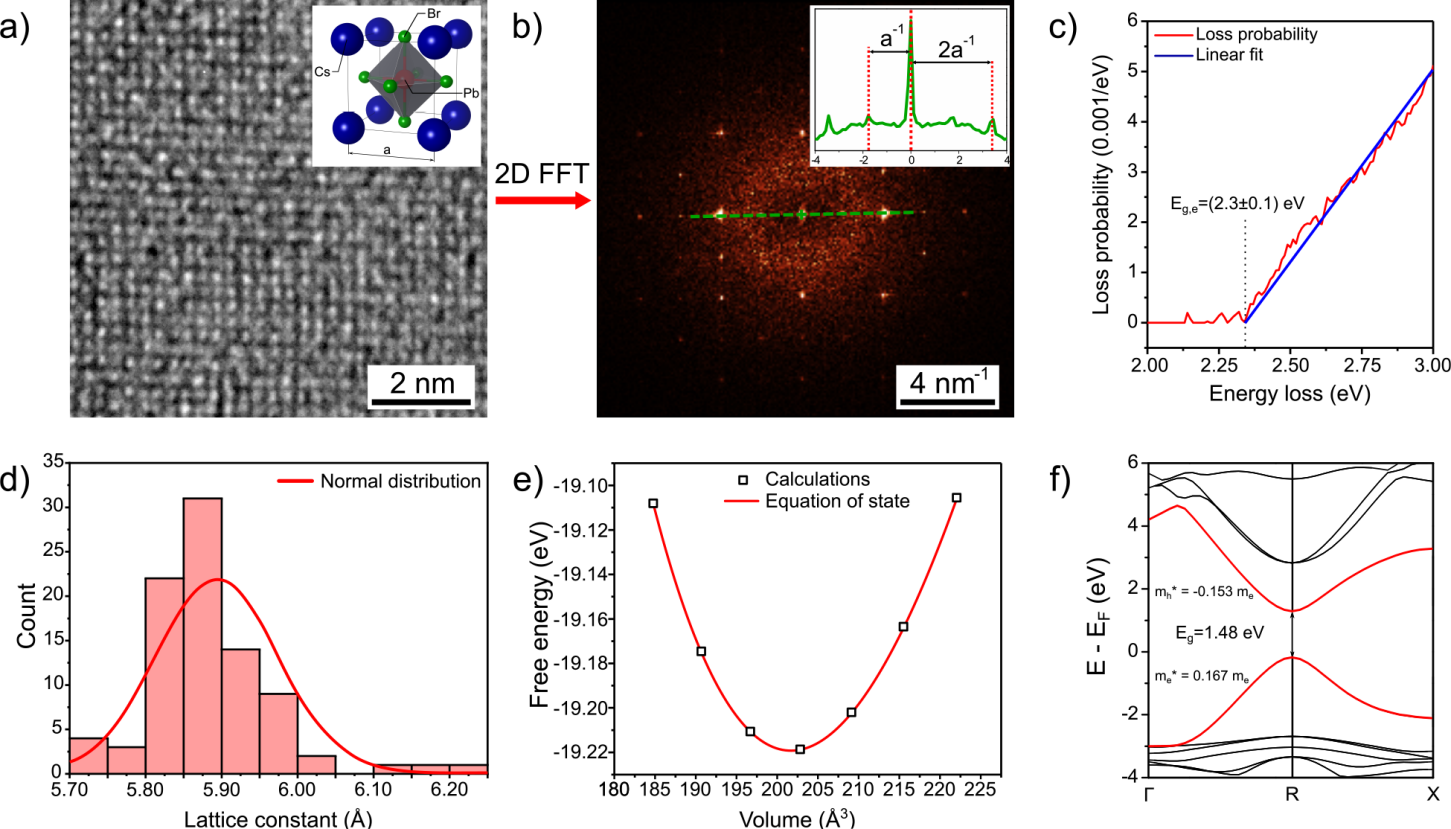
a) Atomic-resolution TEM image of the analyzed CsPbBr_3_ NC with the model of the CsPbBr_3_ cubic lattice. The high-resolution images postprocessed in program Gwyddion^63^ by 2D-FFT to acquire the experimental lattice constant *a*. c) EELS loss probability spectrum of the CsPbBr_3_ NCs with the band gap determined experimentally as *E*_g,e_ = (2.3 ± 0.1) eV. d) A histogram of lattice constants determined for 53 distinct CsPbBr_3_ NCs fitted by normal distribution with the value of experimental lattice parameter *a* = (5.9 ± 0.1) Å. e) Volume relaxation calculations utilizing the experimental lattice parameter *a* as a starting point, fitted by Murnaghan equation of state with an outcome of theoretical equilibrium lattice parameter *a*_0_ = 5.86 Å. f) The theoretically calculated band structure of bulk crystal CsPbBr_3_ acquired via DFT with an input parameter of theoretical equilibrium lattice parameter and output parameters of electron and hole effective masses for effective mass modeling of CsPbBr_3_ NCs’ PL response.

To predict the influence of the CsPbBr_3_ NCs’ morphology on their PL properties, we built a quantum confinement model based upon high-resolution TEM measurements and effective mass theory with parameters obtained by DFT. Figure 4a displays atomic-resolution TEM images of the CsPbBr_3_ NCs prepared by the hot-injection method, measured in order to obtain the lattice parameter of the NCs. We were unable to directly observe the lattice parameter in the NCs presented in the correlative imaging since they degraded quickly while exposed to the high-energy electron beam (see Supporting Information S1, Figure S2). The atomic-resolution images of CsPbBr_3_ NCs were then processed by a 2D fast Fourier transform (2D FFT), shown in Figure 4b, in order to determine the lattice constant. To determine the experimental value of the CsPbBr_3_ band gap, electron energy loss spectroscopy (EELS) measurements took place (Figure 4c), which indicated the band gap of CsPbBr_3_ to be *E*_g,e_ = (2.3 ± 0.1) eV. The experimentally obtained lattice parameters from several NCs were displayed in a histogram (Figure 4d) that was fitted by a normal distribution. The statistically determined value of the lattice parameter was determined to be *a* = (5.9 ± 0.1) Å, which is in good agreement with the value *a* = 5.865 Å^62^ reported in the literature.

Figure 4e displays DFT calculated free energies as the function of the unit cell volume and a fit to the Murnaghan equation of states (eq 2). The equilibrium lattice constant of CsPbBr_3_ was found to be *a*_0_ = 5.864 Å, which agrees well with the experiment. Figure 4f displays the band structure of bulk CsPbBr_3_ calculated by DFT with the equilibrium lattice constant as an input. Due to the nature of the DFT calculations, which artificially reduce the value of the band gap, the theoretical value of the band gap was determined as *E*_g,t_ = 1.48 eV. For the purpose of the QCE model in this paper, we have used the measured value of the band gap by EELS *E*_g,e_ = (2.3 ± 0.1) eV as a starting point for the consequent QCE simulations. From the band structure, the electron and hole effective masses *m*_e_^*^ = 0.167 *m*_e_, and *m*_h_^*^ = −0.153 *m*_e_ in the direction Γ–R were obtained, where *m*_e_ is the mass of a free electron. These values are in good agreement with ref (25); however, the incorrect value of the band gap suggests that these effective masses can vary by order of several percent.

**Figure 5:**
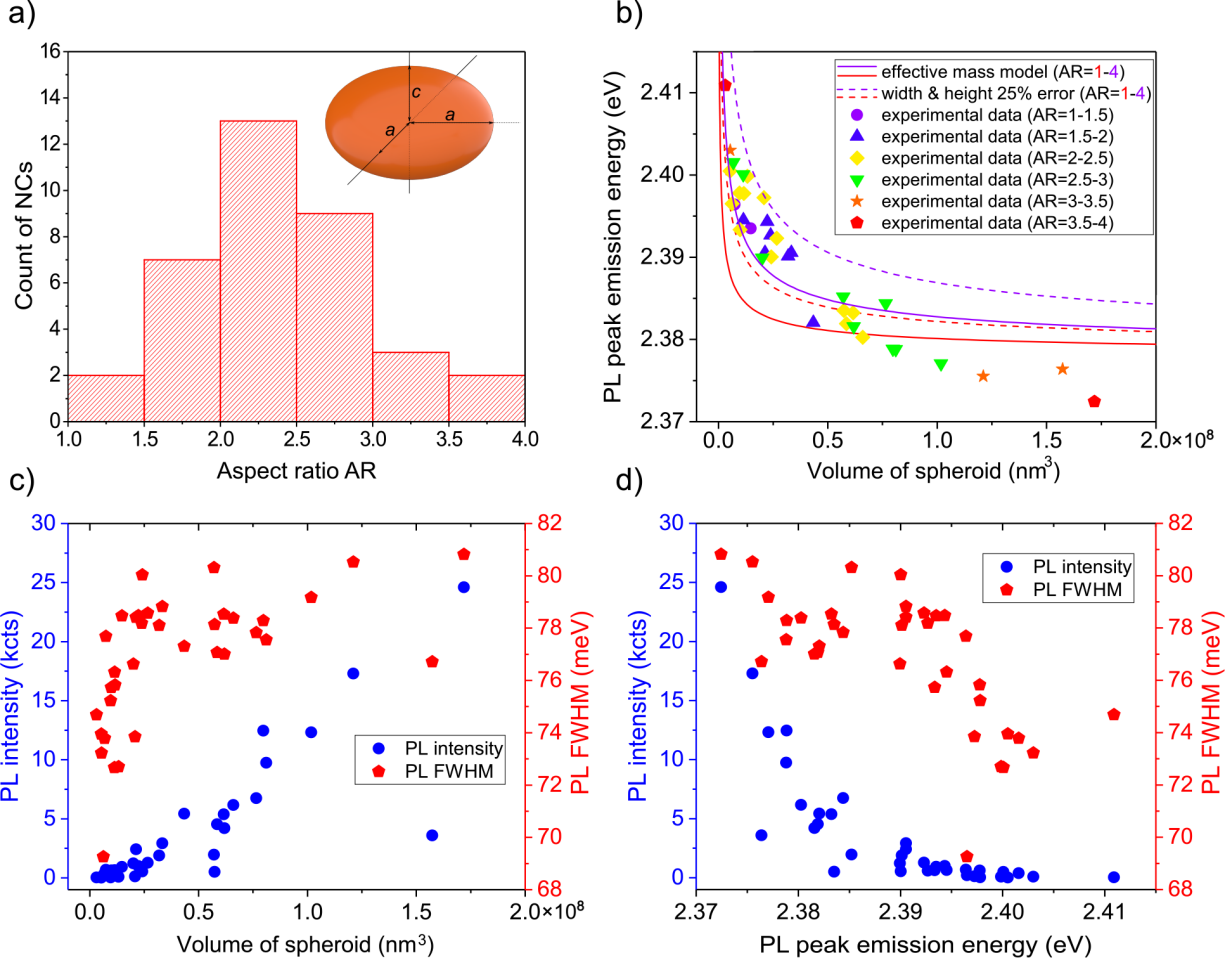
a) Histogram of the NCs aspect ratio *a*/*c* ranging between 1 and 4 with the 3D model of a spheroid used to represent the NCs by the main axis parameters *a* and *c*. b) The experimentally obtained PL peak emission energies of individual CsPbBr_3_ NCs of particular aspect ratios are plotted as a function of the spheroid volume and compared to the QCE models predicting the volume-and-aspect-ratio-dependent PL peak energy shift. The dashed lines correspond to the assumption that the effective dimensions of NCs are by 25% smaller than those determined from SEM and AFM images. The PL intensity and PL fwhm have been plotted as the functions of c) volume of the NCs and d) PL peak emission energy.

For the purpose of predicting the dependency of the CsPbBr_3_ NCs’ PL response within effective mass theory, we have proposed a model shape of NCs as a spheroid with only two parameters: the volume and the aspect ratio. Even though this shape does not fully correspond to the real image of the CsPbBr_3_ NCs, it respects the real aspect ratios. We were able to obtain the aspect ratio *AR* = *a*/*c*, where *a* is the length of the NCs (Figure 1a) and *c* is the height of the NCs (Figure 1b) from the CsPbBr_3_ NCs’ morphology. In Figure 5a, the histogram of the aspect ratios of individual NCs with values ranging from 1–4 is shown, with the values 2–2.5 being the most common. This means that the lateral dimensions of the CsPbBr_3_ NCs tend to be larger than the vertical dimensions. The total volume of a such spheroid is then calculated as .

Let us consider an infinite potential well with the shape of a spheroid representing a single CsPbBr_3_ NC. The energy level distribution for an exciton confined within this system can be expressed as
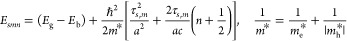
4where *E*_g_ = 2.425 eV is the band gap of CsPbBr_3_, m* = 0.08*m*_e_ is the reduced effective exciton mass, *E*_b_ = 47 meV is the binding energy of an exciton accounting for the Coulomb interaction between an electron and a hole (see Methods), τ_*s*,*m*_ is the *s*th root of the Bessel function of the first kind where *s* = (0, 1, 2, ...), *m* = (1, 2, 3, ...), and *n* = (0, 1, 2, ...) as described in ref (64). The experimentally and theoretically retrieved values for the PL peak emission energy of individual NCs are plotted and compared as a function of spheroid volume with a varying aspect ratio in Figure 5b. The theoretical values are systematically somewhat larger than the experimental ones. A plausible explanation is that the effective dimensions of NCs are smaller than the dimensions determined from the SEM and AFM images. Indeed, the wave function needs to vary smoothly in space. In the realistic NCs of irregular shape, the wave function cannot exploit the full volume of the particle. Instead, it approximately takes a spheroidal shape inscribed to the NC (see e.g. Figure 4 in ref (65)). Consequently, the effective dimensions of the NCs are smaller. By analysis of the realistic particle shapes, we estimate the difference of the effective and real dimension to be up to 25% (see Supporting Info S2, Figure S3). We show the corresponding theoretical dependences assuming the effective dimensions of NCs by the dashed lines in Figure 5b. Now, most of the experimental points agree well with this corrected theoretical prediction. It is also notable that the amplitude of the PL peak emission energy shift (∼39 meV/9 nm) being smaller than the average PL fwhm (∼75 meV/16 nm) results in a uniform macroscopic optical response of the hereby presented statistical ensemble of CsPbBr_3_ NCs across the sample with the PL peak emission energy shift detected only by high-resolution optical imaging.

In Figure 5c, a comparison is made between the experimentally determined PL intensity, PL fwhm, and NCs’ volume. Importantly, the PL intensity exhibits a linear dependence on the particle volume with 75% of NCs having a deviation from the linear relationship below 20% (blue points in Figure 5c). The effect of nonradiative processes, which could have significantly decreased the intensity of small particles on the surface, is insignificant. Notably, only 11% of the particles show a considerably lower PL intensity, which can be attributed to structural defects, lattice strain, presence of the trap states, or other nonradiative recombination channels.^66,67^ Consequently, the total emission intensity is primarily linked to the volume of the material, indicating that the grain size does not have a significant impact. In Figure 5d, a comparison is made among experimentally determined PL intensity, PL fwhm, and PL peak emission energy. The PL intensity significantly decreases as the PL peak emission energy increases. The fwhm of the emission exhibits a slight but systematic increase for larger particles. At present, the origin of this effect is unknown to us. We note that for very small particles (several units of nm), the opposite trend has been observed previously and attributed to the size dependence of the phonon–exciton coupling strength.^35^ For spectroscopic purposes, we display these results in the units of nanometers in the Supporting Info S3, Figure S4.

## Conclusions

In conclusion, we performed a comprehensive analysis of CsPbBr_3_ NCs at an individual NC level. It consisted of correlative high-resolution morphological and optical analysis, which has been used to determine the relation between the shape and dimensions of NCs and their PL peak emission energy. These experimental results have been used as input to a simplified QCE model based on the effective mass approach. We were able to successfully predict the PL peak emission energy for CsPbBr_3_ NCs based on the model of an exciton confined within the infinite spheroidal potential well. Our correlative analysis of a large statistical ensemble of individual CsPbBr_3_ NCs has the following implications for their utilization in various optoelectronic applications: (i) CsPbBr_3_ NCs produced by the simple drop-casting method are of high quality, exhibiting bright and size-tunable PL emission. (ii) The simplified and aspect-ratio-based QCE model with effective masses obtained from DFT calculations qualitatively agrees with observed PL peak emission energies of CsPbBr_3_ NCs without the need for complex numerical simulations. Despite relatively big variations in the shape and size of individual NCs, the observed QCE is moderate, up to 9 nm in the wavelength. This is considerably smaller than their average PL fwhm, thus being detectable only by high-resolution PL mapping. (iii) The PL intensity exhibits a linear dependence on the particle volume, and thus, the total PL emission intensity of the NCs is primarily linked to the volume of the material, indicating that the grain size does not have a significant impact. (iv) CsPbBr_3_ NCs are compatible with the FIB processing with Ga ions, which poses an opportunity for precise nanoprocessing of individual NCs as well as their integration into more complex devices including optical cavities or waveguides.

## Supporting Information Available

Sensitivity of CsPbBr_3_ NCs toward FIB and high-energy electron beam in TEM; suitability and error of NCs spheroidal approximation; results achieved by correlative imaging in spectroscopic units (PDF)
