# Supplementary material for: Correlative Imaging of Individual CsPbBr3 Nanocrystals: Role of Isolated Grains in Photoluminescence of Perovskite Polycrystalline Thin Films
Source: J Phys Chem C Nanomater Interfaces. 2023 Jun 20;127(25):12404–13. doi: 10.1021/acs.jpcc.3c03056 (PMC10316395; doi:10.1021/acs.jpcc.3c03056)
Supplement: Supplementary file 1 — jp3c03056_si_001.pdf [file jp3c03056_si_001.pdf]

Supporting information: Correlative Imaging of Individual $CsPbBr_3$ Nanocrystals: Role of Isolated Grains in Photoluminescence of Perovskite Polycrystalline Thin Films

Petr Liška,[*,†,‡] Tomáš Musálek,[†] Tomáš Šamořil,[†,‡,§] Matouš Kratochvíl,[¶] Radovan Matula,[†] Michal Horák,[†,‡] Matěj Nedvěd,[†] Jakub Urban,[†] Jakub Planer,[‡] Katarína Rovenská,[‡] Petr Dvořák,[†,‡] Miroslav Kolíbal,[†,‡] Vlastimil Křápek,[†,‡] Radek Kalousek,[†] and Tomáš Šikola[†,‡]

†*Institute of Physical Engineering, Faculty of Mechanical Engineering, Brno University of Technology, Technická 2896/2, 616 69 Brno, Czech Republic*

‡*Central European Institute of Technology, Brno University of Technology, Purkyňova 123, 612 00 Brno, Czech Republic*

¶*Faculty of Chemistry, Brno University of Technology, Purkyňova 464/118, 612 00 Brno, Czech Republic*

§*TESCAN ORSAY HOLDING, a.s, Libušina tř. 21, Brno 623 00, Czech Republic*

E-mail: petr.liska1@vutbr.cz

S1: Sensitivity of $CsPbBr_3$ nanocrystals to FIB and electron beam in TEM

The exposure to the required dose of Ga ions during the fabrication of FIB micro-markings decreases the PL integral intensity by about 6 % (Figure S1) but does not alter the PL peak emission wavelength or FWHM.

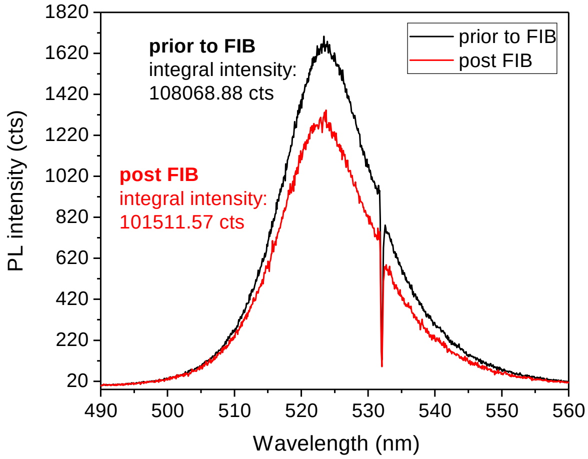

Figure S1: Prior to FIB processing and post FIB processing obtained typical PL integral intensities of $CsPbBr_3$ NC.

Due to the intense destruction of $CsPbBr_3$ NCs during the TEM analysis (Figure S2a), which resulted in the incapability of atomic-resolution images nor diffraction patterns, a compromise needed to be made. The acquisition of the TEM and EELS measurements has been made on the colloidal $CsPbBr_3$ NCs, which should exhibit the same crystalline structure as the $CsPbBr_3$ NCs prepared by drop casting. The STEM overview image of the colloidal nanocrystals is displayed in Figure S2b. The challenges of the $CsPbBr_3$ NCs and thin films imaging have been already discussed in[1,2] which results are in agreement with our observations.

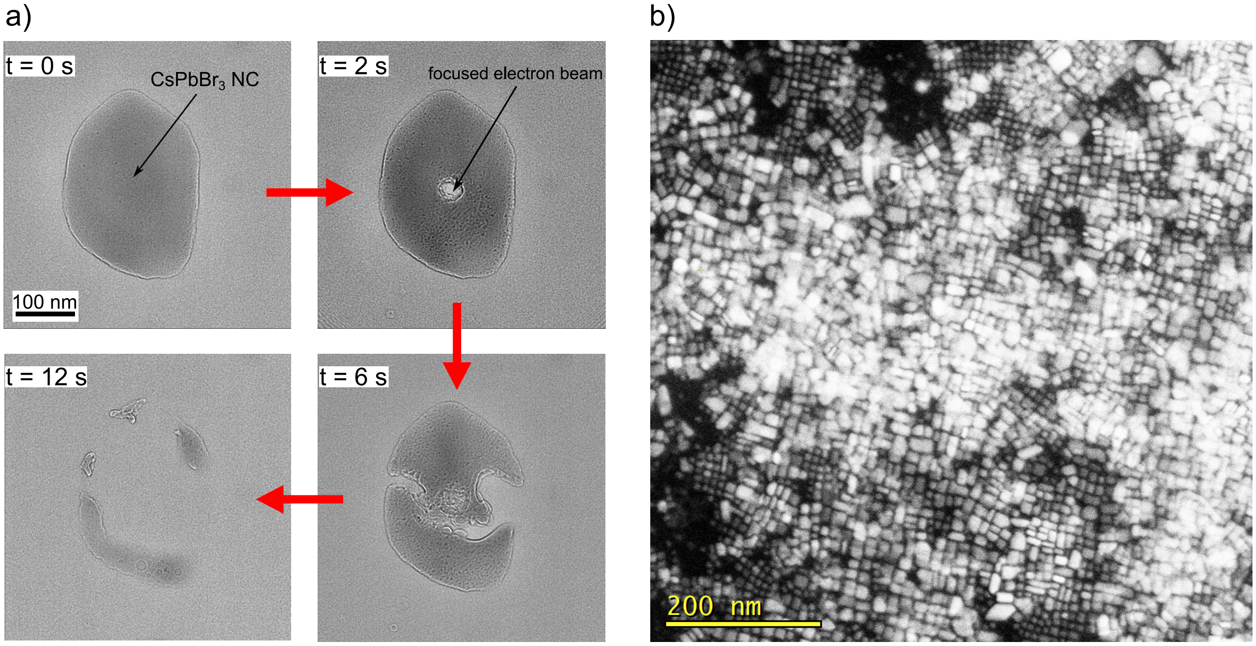

Figure S2: a) Immediate degradation of drop casted $CsPbBr_3$ NCs in TEM observation under focused electron beam. b) Overview STEM image of colloidal $CsPbBr_3$ NCs serving for the crystal lattice acquisition.

S2: Error of the spheroidal approximation

In order to estimate the error of the used spheroid approximation of the $CsPbBr_3$ NCs, the effective length a and height c of the NC have been compared to the lengths and heights of the inscribed $a_{\mathrm{in}}, c_{\mathrm{in}}$ (Figure S3a) and circumscribed $a_{\mathrm{out}}, c_{\mathrm{out}}$ (Figure S3b) spheroid. The effective lengths a have been acquired from each NC by averaging over 12 px from the horizontal profile in SEM and effective heights c have been acquired by averaging over 12 px from the topography of individual NC in the program Gwyddion.[3] The lengths and heights of the inscribed or circumscribed spheroid were obtained by fitting the spheroid inside the NC or fitting the spheroid so it would contain the whole NC respectively. Figure S3 displays the comparison between the a) inscribed or b) circumscribed lengths and heights of the NCs with the effective values. The average deviation of the used values and the effective ones was determined ranging between $5 - 25\,\%$.

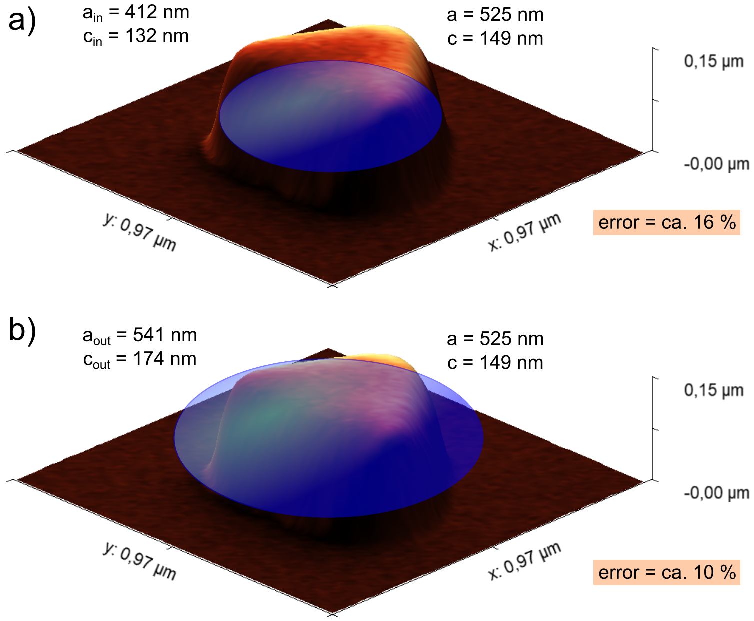

Figure S3: a) Inscribed and b) circumscribed spheroid compared to the effective length and height of the NC.

S4: Optical properties of CsPbBr3 NCs in spectroscopic units

For purposes of optical spectroscopy, we include Figure 3 from the main manuscript also in the units of wavelengths (Figure S4).

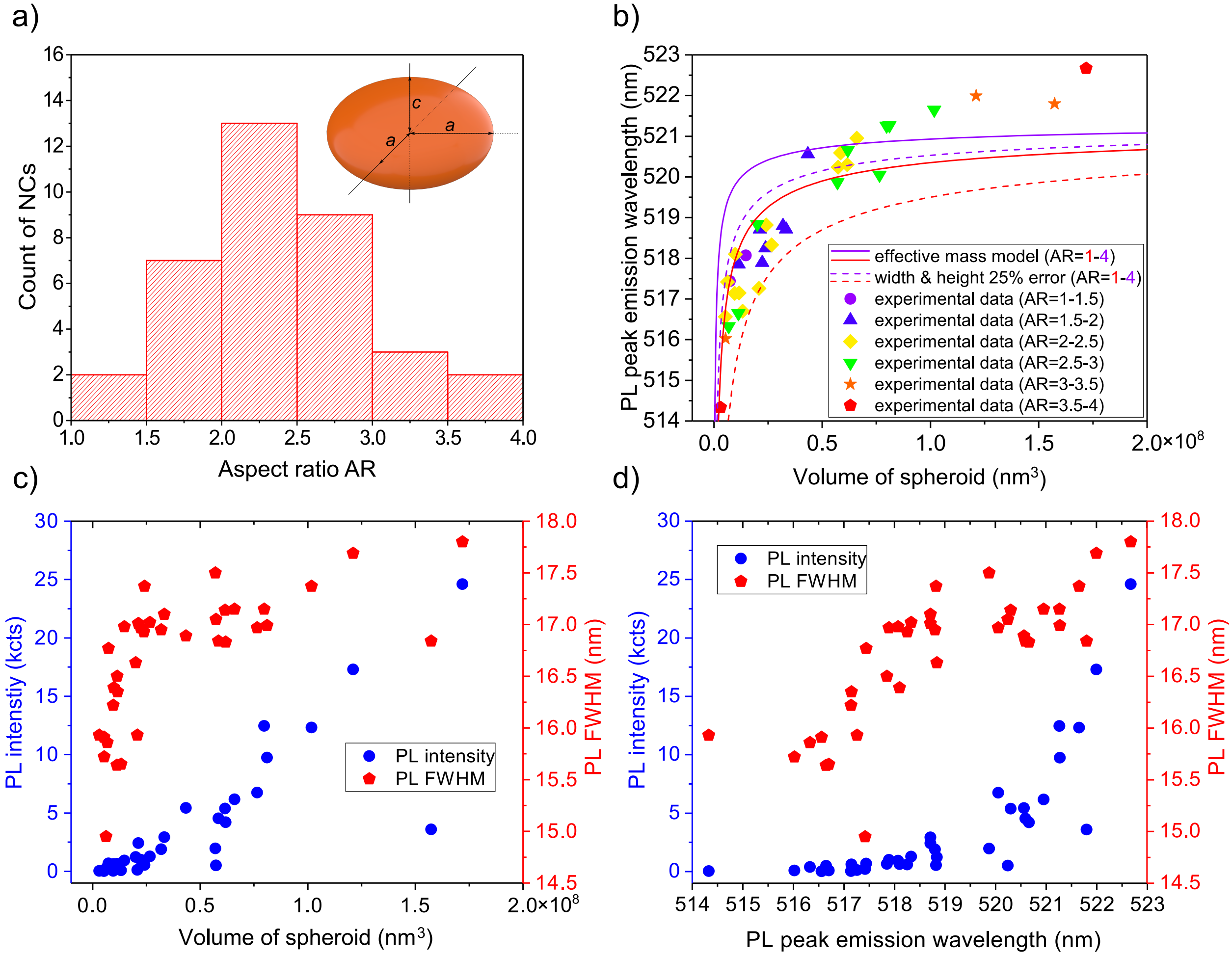

Figure S4: a) Histogram of the NCs aspect ratio a/c ranging in between 1 and 4 with the 3D model of a spheroid used to represent the NCs by the main axis parameters a and c. b) The experimentally obtained PL peak emission wavelengths of individual $CsPbBr_3$ NCs of particular aspect ratios are plotted as a function of the spheroid volume and compared to the QCE models predicting the volume-and-aspect-ratio-dependent PL peak wavelength. The dashed lines correspond to the assumption that the effective dimensions of NCs are by 25 % smaller than those determined from SEM and AFM images. The PL intensity and PL FWHM have been plotted as the functions of c) volume of the NCs and d) PL peak emission wavelength.

References

(1) Brennan, M.; Kuno, M.; Rouvimov, S. TEM Analysis of $CsPbBr_3$ Nanocrystals. *Microscopy and Microanalysis* **2017**, *23*, 2096–2097.

(2) Duong, T. M.; Sharma, K.; Agnese, F.; Rouviere, J.-L.; Okuno, H.; Pouget, S.; Reiss, P.; Ling, W. L. Practice of electron microscopy on nanoparticles sensitive to radiation damage. *Frontiers in Chemistry* **2022**, *10*, 1058620.

(3) Nečas, D.; Klapetek, P. Gwyddion: an open-source software for SPM data analysis. *Central European Journal of Physics* **2012**, *10*, 181–188.
